# Simultaneous atomic-level visualization and high precision photocurrent measurements on photoelectric devices by *in situ* TEM†

**DOI:** 10.1039/c7ra10696c

**Published:** 2018-01-03

**Authors:** Hui Dong, Tao Xu, Ziqi Sun, Qiubo Zhang, Xing Wu, Longbing He, Feng Xu, Litao Sun

**Affiliations:** SEU-FEI Nano-Pico Center, Key Laboratory of MEMS of the Ministry of Education, Southeast University Nanjing 210096 China fxu@seu.edu.cn; School of Chemistry, Physics and Mechanical Engineering, Queensland University of Technology Gardens Point Brisbane QLD 4000 Australia; Southeast University-Monash University Joint Research Institute Suzhou 215123 P. R. China slt@seu.edu.cn; Department of Electrical Engineering, East China Normal University 500 Dongchuan Road Shanghai 200241 China

## Abstract

Herein, a novel *in situ* transmission electron microscopy (TEM) method that allows high-resolution imaging and spectroscopy of nanomaterials under simultaneous application of different stimuli, such as light excitation, has been reported to directly explore structure–activity relationships targeted towards device optimization. However, the experimental development of a photoelectric system capable of combining atomic-level visualization with simultaneous electrical current measurement with picoampere-precision still remains a great challenge due to light-induced drift while imaging and noise in the electrical components due to background current. Herein, we report a novel photoelectric TEM holder integrating an LED light source covering the whole visible range, a shielding system to avoid current noise, and a picoammeter, which enables stable TEM imaging at the atomic scale while measuring very small photocurrents (pico ampere range). Using this high-precision photoelectric holder, we measured photocurrents of the order of pico amperes for the first time from a prototype quantum dot solar cell assembled inside a TEM and obtained atomic-level imaging of the photo anode under light exposure. This study paves the way towards obtaining mechanistic insights into the operation of photovoltaic devices by providing direct information on the structure–activity relationships that can be used in device optimization.

## Abstract 2

A photoelectric system is capable of simultaneous atomic-level visualization and pico-ampere-precision.

## Graphical abstract

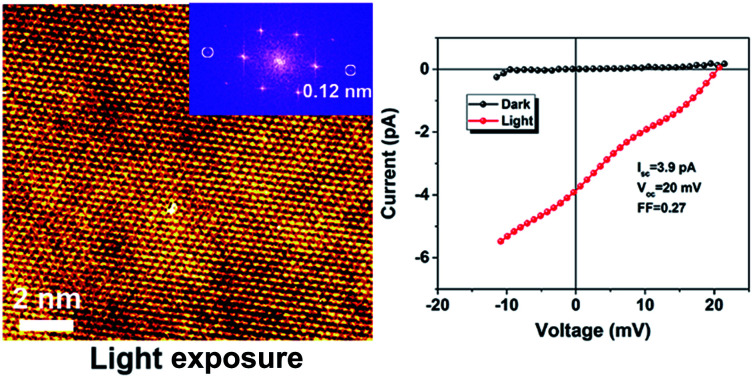

## Introduction

*In situ* transmission electron microscopy (TEM) encompasses a broad range of techniques where high-resolution imaging and spectroscopy are carried out while applying various stimuli to the sample material. Recent studies mainly focus on the application of a single stimulus, which may be heat, stress, electrical biasing, or ultrashort photon pulses, on the material.^1^ By applying electric fields on a ferroelectric architecture in operation, Pan's group directly observed the kinetics and dynamics of electric domain switching and how the defects and interfaces impede full ferroelectric switching of a thin film.^2^ Using *in situ* TEM, Gao *et al.* reported the dynamic observation of the growth of a conducting filament in nanoscale resistive memories and oxygen vacancy migration resulting in electrically induced change in resistance in cerium oxides.^3,4^ A nanoscale electrochemical device was assembled inside a TEM to observe the lithiation behaviour of a nanowire *in situ* during electrochemical charging, which served to advance the fundamental understanding of the electrochemical reactions and the mechanism of degradation of the material during operation.^5^ Inspired by the success of *in situ* TEM technology in combination with electrical and electrochemical analysis, photocatalytic reactions were monitored inside a TEM to understand the structure–activity correlations in the photocatalyst during a catalytic reaction while introducing light as a stimulus.^6^ Light-illumination sample holders have also been designed for environmental TEM (ETEM) to investigate the photodecomposition of a photocatalyst such as the transformation of Cu_2_O to Cu under UV irradiation in a H_2_O environment.^7^ A fibre-based photoexcitation system for ETEM, which allows to control the temperature, gas environment, and light, has been developed by the Crozier's group, and this photoexcitation system has been used to investigate the changes taking place on the surface of anatase nanocrystals during photo-induced splitting of water molecules in the vapor state.^8^ These studies contribute towards an in-depth understanding of the structure–activity correlations at the atomic level. However, often, the information about the relationship between the structure and functionality is incomplete while using only a single-field as a stimulus. In the study on photoelectric devices, the major challenge is to directly correlate the observed light-induced current with local structural in-homogeneities and dynamics.^9^

Novel *in situ* tools to provide multi-field coupling conditions have, therefore, been recently developed to accurately characterize the properties of individual devices in real time.^10^ The electrical transport coupled with optical and piezoelectric properties of individual ZnO nanowires was studied *in situ* by introducing ultraviolet illumination during TEM imaging.^11^ The photoelectric performances of individual CdS nanowires and CdS/ZnO heterostructures were characterized using a novel laser-compatible high-resolution *in situ* TEM technique.^12,13^ These findings provide mechanistic insights into how strain affects the photoelectric properties of materials and offer guidelines for the design of future flexible electronic, optoelectronic, and photovoltaic devices. However, the power conversion efficiency and long-term performance of photovoltaic devices and photodetectors are still very low, and there are many fundamental problems that need to be solved to further improve their performance. It is now recognized that atomic level *in situ* observation of materials is critical to relate the structure of photoelectric devices to their performance. To date, there is no multimodal optical-electric system that can directly correlate the atomic structure with photoelectric performance because of two reasons: the introduction of light introduces considerable drift in TEM imaging, and the noise in the electrical circuit is detrimental to making precise photocurrent measurements.

Herein, we report a multimodal photoelectric TEM sample holder capable of imaging materials at the atomic-scale while simultaneously measuring photocurrents with pico ampere precision. This *in situ* photoelectric holder enables to use light sources with different wavelengths and also ensures ultrasensitive pico ampere-precision measurement without compromising the microscope's performance, especially with respect to imaging at the atomic scale. Examples of these mutually compatible measurements include use of a custom-designed photodetector with a quantum dot sensitized solar cell while successfully imaging the changes in its structure under light exposure at the atomic level inside a TEM. Of particular technological importance is the capability to have a fundamental understanding of the structure and photoelectric performance at the atomic level and provide specific guidelines for the design of high-efficiency photoelectric devices.

## Experimental

### Sample preparation

ZnO nanowires (NWs) were prepared using a simple hydrothermal method similar to that previously reported by Cheng Bin *et al.*^14^ Mercaptopropionic acid (MAP)-capped CdSe QDs in an aqueous solution were prepared using previously reported procedures.^15^ The NWs were immersed in a solution of QDs for 2 h after which, they were rinsed sequentially with water and ethanol. The precipitate of ZnO NW/CdSe QDs was dried and stored for further *in situ* TEM experiments. CdS nanoparticles were prepared by a simple successive ionic layer adsorption and reaction technique,^16^ and the precipitated powder was obtained and used for *in situ* characterization.

### Assembling quantum dot-sensitised solar cells (QDSSCs) and a CdS nanoparticle photodetector inside a TEM

In our *in situ* experiment, the CdS and CdSe QD-coated-ZnO NWs were mounted on a copper electrode by immersing a copper grid in the corresponding solution in ethanol. The movable metallic electrode is a W and Pt tip, controlled by a piezo-tube to make contact with the CdS and CdSe QD-coated ZnO NW mounted on the copper grid. The photoelectric properties of the individual nanowire QDSSCs were measured inside the TEM (Titan 80-300, FEI) using a custom-made Nanofactory *in situ*-scanning tunneling microscope (STM)-TEM electrical probe, which was modified to incorporate a white LED to function as a light source. A pA Keithley 6430 source meter was used to measure the solar cell performance by tracing the photocurrent *vs.* time and current–voltage curves. Before each experiment, we used an irradiance meter (FGH-1, photoelectric instrument factory of Beijing Normal University) to calibrate the light source by slightly tuning the input voltage for obtaining the same light intensity.

## Results and discussion

**Fig. 1:**
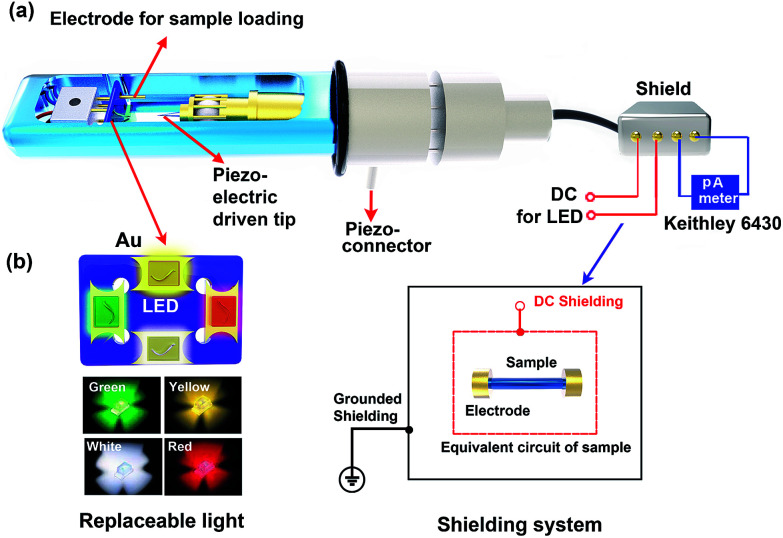
(a) The schematic of our indigenous photoelectric sample holder. The LED driven by DC power is placed on the sapphire substrate, which is inserted into the slot previously occupied by the electrical measurement system in the STM-TEM. A Keithley 6430 pA meter is used to obtain the photocurrent following excitation by photo irradiation from the LED. (b) A schematic of the replaceable LED and design of the shielding system.

The custom-designed photoelectric TEM holder combined with an external electric measurement system designed based on the commercially available Nanofactory: TEM-STM holder is shown in Fig. 1a. The homemade *in situ* photoelectric set up including a light-emitting diode (LED) as the light source and the electrode tip for sample loading replaces the electrical measuring system of the STM holder; the shielding system connected with a pico ampere meter is designed to measure ultra-small currents. The process used to deposit the LED on sapphire (Al_2_O_3_) is represented in Fig. S1.† As schematically depicted in Fig. 1b, different kinds of LEDs, for example, white, red, yellow, and green, can be placed on the sapphire substrate, and these can be interchanged expediently by implanting different LEDs on the substrate. To reduce noise during current measurement, a shielding system consisting of a grounded shielding and a direct current (DC) shielding was designed, as shown in Fig. 1b. The equivalent circuit of the sample shows that it can be considered to be protected by both DC shielding and grounded shielding, which enables the detection of ultra-small photocurrents stimulated by light.

**Fig. 2:**
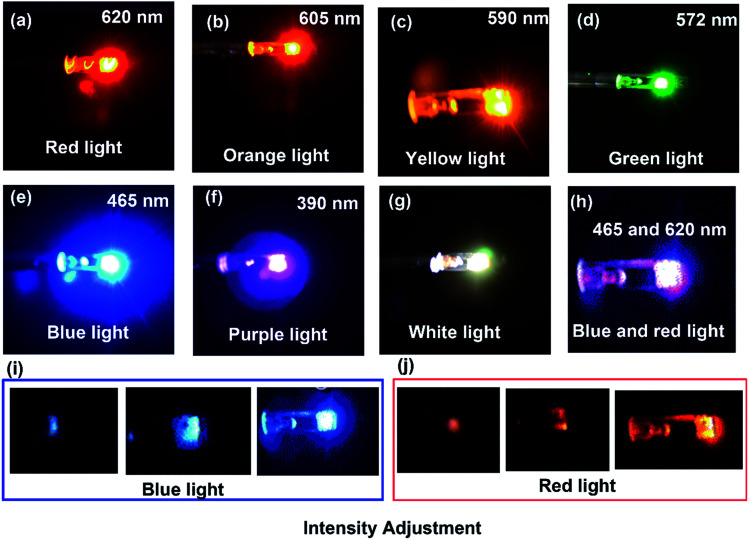
Visible light emission of different wavelengths from LEDs implanted on the holder inside the TEM. (a–g) Show red, orange, yellow, green, blue, purple, and white emission. (h) Both red and blue light can be switched on simultaneously. The intensity adjustment of blue (i) and red light (j).

Fig. 2 shows the images of light emission of different wavelengths obtained by the incorporation of different LEDs on the holder for *in situ* TEM. The actual image of the photoelectric holder with the blue-light-on is shown in Fig. S2.† Red, orange, yellow, green, blue, and purple light, with the wavelengths of 620, 605, 590, 572, 465, and 390 nm, respectively, can be successfully placed on the holder, as shown in Fig. 2 a–f. In addition, the switching on and off of the light source can be controlled internally, as demonstrated in Movie S1,† taking blue light as an example. We have also implanted white light on the holder, as shown in Fig. 2g. Interestingly, two kinds of light with different wavelengths, for example, both blue and red light (Fig. 2h), can be simultaneously implanted on the holder (please see Movie S2†). Moreover, the intensity of light can be adjusted by regulating the output voltage of our homemade power supply system (Fig. 2i and j), Movies S3 and S4,† respectively, show how the blue and red light intensities can be adjusted. This enables us to explore structure–reactivity relations in the photosensitive material under light of different energies and intensities. Although light and electron beams interact with specimens in a similar way, the energy of the electrons is higher and can therefore induce effects, which are absent under light illumination.

**Fig. 3:**
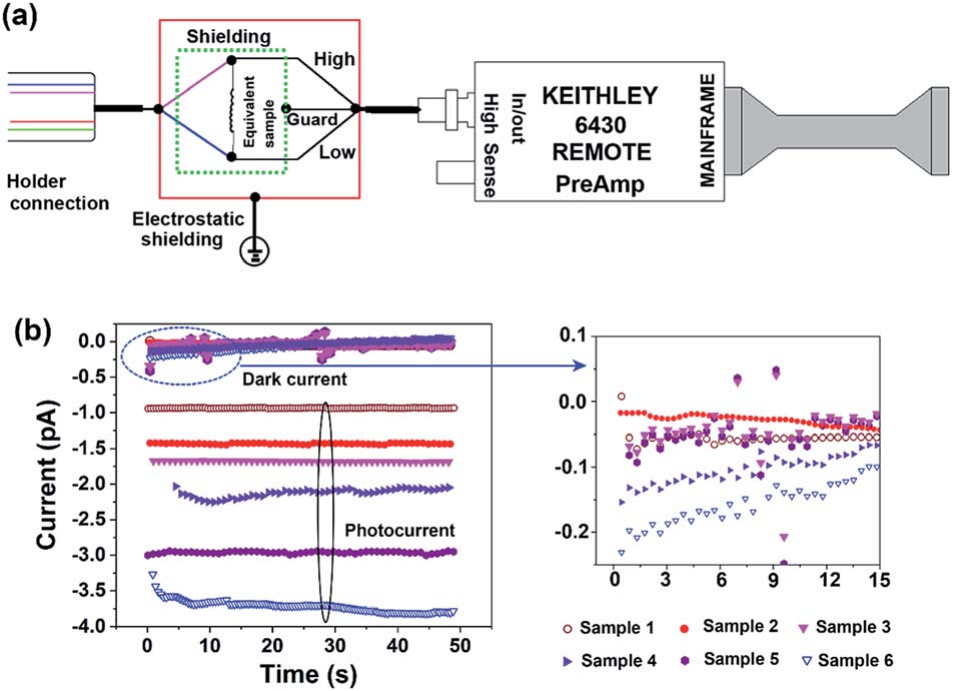
(a) The diagram of the current measurement and the shielding system. (b) Current–time curves in the dark and under light exposure. Using our indigenous shielding system, photocurrents in the picoampere range can be detected.

The circuit diagram for the photocurrent measurement in the shield is shown in Fig. 3a. In the Keithley model 6430 sub-femto-amp remote source meter, the triax connector consists of a central conductor with a connector (high) and an outer shell (low) for electrical measurements and an inner shell (guard), which, while acting as a DC shield, is used to reduce noise. Thus, the guard port is connected with the inner shield such that to have the same potential as the high port, and no current leakage occurs from the sample. In the outer layer, an electrostatic shielding with grounding is necessary to eliminate the influence of electric and magnetic fields present in the environment. Upon combining the two shield systems, current measurements with 0.5 pA accuracy under dark conditions could be achieved; (multiple measurements are performed for each sample to ensure reproducibility), and light-stimulated photocurrent at the picoampere level can be detected (Fig. 3b). In conclusion, the designed system is suitable for ultra-small current measurements and enables the correlation of the crystal structure with device performance.

**Fig. 4:**
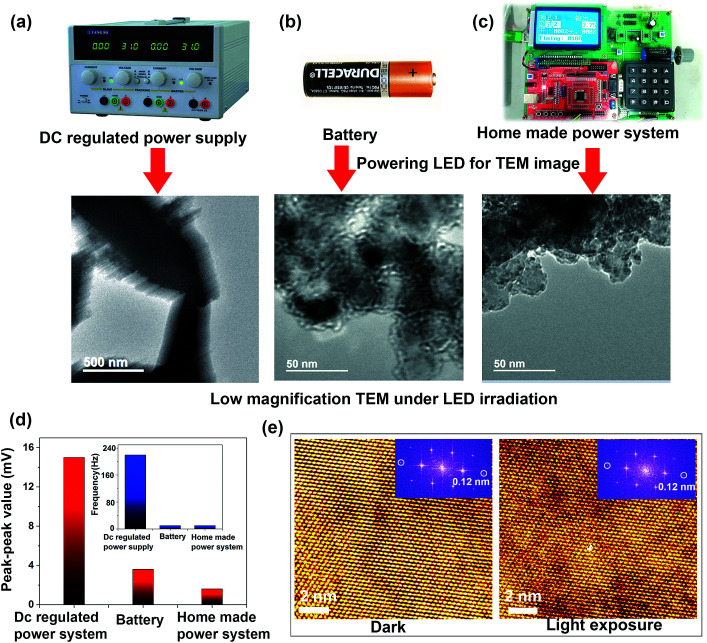
Low-magnification TEM image obtained under light irradiation from LED powered by DC-regulated power supply (a), battery (b), and our custom-designed homemade power supply system (c). The precise control of voltage of our homemade power system allows the application of voltages as low as 0.01 V. (d) The peak–peak value of the output voltage and frequency for different power systems measured by oscillography. (e) HRTEM image of the ZnO nanowire in the dark and under light exposure using our custom-designed power supply system driving the LED.

In Fig. 4a–c, low-magnification TEM images obtained under light irradiation from a LED powered by a commercial DC-regulated power supply, a battery, and our custom-designed power supply system, respectively, are shown. We used 220 V AC to drive the DC-regulated power supply, and the AC power is grounded to the same ground as the microscope, such that to eliminate the effects of using different grounds. It is seen that use of a commercial DC-regulated power supply for powering the LED is highly detrimental to the quality of the TEM image, and the image is blurred (Fig. 4a). If we replace the DC-regulated power with a commercial battery, a much clearer image is obtained, but the image is not stable, as shown in Fig. 4b. The bad quality of the TEM image while using the DC-regulated power supply or the battery originates from the unstable output voltage. To circumvent this problem, we designed a stable DC output voltage system composed mainly of five parts (Fig. S3a†): a commercial DC power supply as the power source, an ultra-low-dropout chip TPS7A7001 to provide a steady voltage, a keyboard for the voltage output time setting, an LCD12864 display module to read out the voltage value, and a voltage regulator to adjust the voltage. The code S1 in the ESI† provides the program code in C language that is used to control the power supply system and regulate the output voltage. The working of this system is demonstrated in Fig. S3b.† Movies S5 and S6† represent the TEM image videos under LED light powered by a battery and our home-made power system, respectively. The TEM image illuminated by the battery-powered LED shows a more serious drift as compared to that obtained when our home-made power supply is used; the image is brought immediately to over-focus if adjusted correctly, as shown in Fig. 4b; however, because of the drift, a clearer image than that given in Fig. 4b cannot be obtained. In contrast, using our home-made power system to drive the LED, we could obtain the clearest and most stable TEM image under light exposure (Fig. 4c). The difference in drift is due to the magnetic field brought about by a variation in the electric field due to the much higher peak and the peak value of the output voltage. To confirm this, we measured the peak–peak values and frequencies of output voltages from the DC-regulated power supply, battery, and the homemade power system, as demonstrated in Fig. 4d. The peak–peak value and frequency obtained using our home-made power system are much smaller than those obtained using a battery, as well as those obtained using the DC-regulated power supply (Fig. 4d). Thus, an ultra-steady output voltage can be achieved using our custom-designed system, which contributes to the high quality of the TEM images under light exposure. To further confirm whether the effect has an influence on the TEM image at the atomic level, we also tested the HRTEM image of a ZnO nanowire under dark conditions and under light exposure using our power supply system to drive the LED, as shown in Fig. 4e. The image compares well with that obtained under dark conditions, and an HRTEM image at an atomic resolution of 0.12 nm can be achieved under light exposure; the presence of light has no influence on the image quality (Fig. 4e).

**Fig. 5:**
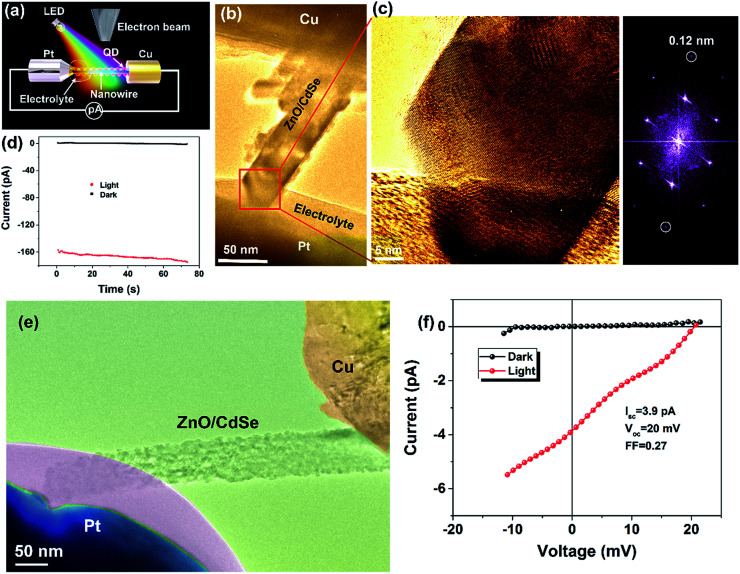
*In situ* fabrication of a photovoltaic device inside TEM. (a) The schematic of *in situ* fabrication of quantum dot-sensitised solar cells. (b) TEM image of quantum dot-sensitised solar cells based on the ZnO nanowire and CdSe quantum dots. (c) An HRTEM image obtained from (b) and the corresponding FFT image obtained under light irradiation. (d) Current–time (*I*–*t*) characteristics under zero bias of the *in situ* fabricated photovoltaic device. (e) TEM image of another ZnO nanowire/CdSe quantum dot-sensitised solar cell. (f) Current–voltage (*I*–*V*) characteristics under dark conditions and under light exposure.

Fig. 5 depicts the application of our home-made photoelectric holder to the construction of a photovoltaic device to be characterized *in situ* inside the TEM. In Fig. 5a, the schematic of a quantum dot-sensitized solar cell (QDSSC) under white light irradiation covering the whole visible light wavelength range consisting of Cu as one electrode, Pt mounted with an ionic liquid electrolyte as the counter electrode, and a nanowire sensitized by QDs as the photo anode, forming a complete QDSSC, is shown. A picoammeter is used to obtain the electric behaviour under both dark and light irradiation of different wavelengths ranging from purple to red. The electron beam is used to obtain a TEM image, with the help of which, a direct correlation of the light-induced electric current to local structural in-homogeneities and dynamics can be obtained. Fig. 5b shows QDSSCs based on a ZnO nanowire and CdSe quantum dots constructed inside the TEM. The three constituents of a standard QDSSCs can be clearly observed, namely, the photoelectrode, electrolyte, and the counter electrode; the ZnO nanowire is sufficiently covered by CdSe QDs. In Fig. 5c, the HRTEM image obtained from Fig. 5b is shown, where a resolution of 0.12 nm can be obtained under light irradiation. We measured current–time curves in the dark and under stimulating illumination, as shown in Fig. 5d. At zero bias, a photocurrent of ∼160 pA is detected while the dark current is ∼0 pA, indicating that the QDSSCs constructed *in situ* in the TEM are operational when irradiated by light (the electron beam is kept at the same intensity, and only the light source is switched on and off). To confirm that the photocurrent originated only due to photo-stimulation, we also characterized the effect of electron beam of different intensities on current measurements under dark conditions (Fig. S4†). At a low electron beam intensity and low magnification, there is an obvious current induced by the electron beam, but this current does not change with time. However, at higher electron beam intensity, or in HRTEM magnification, the current induced by the electron beam can be ignored as compared to that under the beam-off condition. This is mainly because contrary to a low intensity beam, a high intensity electron beam can completely penetrate the sample. Although the electron beam effect can be ignored during HRTEM image capture, we have measured the photocurrent while keeping the intensity of the electron beam constant. This enables us to directly correlate the atomic image to the photocurrent without considering the beam effect at a high magnification in future experiments. With characterising photovoltaic devices, it is the current–voltage (*I*–*V*) response that is the most important parameter. Under real conditions, the operation of a solar cell is under a certain forward bias. To confirm that our homemade holder is capable of biasing the sample under illumination, we obtained the TEM image of the assembled device and measured the *I*–*V* curves under dark and under exposure to light, as shown in Fig. 5e and f. False colour has been used to clearly identify the different parts of the QDSSC, as shown in Fig. 5e, namely, the Cu electrode, Pt mounted with an ionic liquid electrolyte, and the ZnO/CdSe, which together form a complete QDSSC structure. From Fig. 5f, the photovoltaic effect can be clearly observed and the short circuit current, fill factor, and open voltage are 3.9 pA, 0.27, and 20 mV, respectively. A picoammeter combined with double shielding systems can detect ultra-small photocurrent signals at the pA level, whereas the electron beam determined the dynamics of structural variation at the atomic level, which enables us to correlate the atomic crystal structure with photovoltaic performance. This provides an insight into the structure and photoelectric performance at the atomic level and paves the way to designing high-efficiency solar cells as well as other photovoltaic devices. We also fabricated a photodetector device based on nanoparticles, as shown in Fig. S5.† Fig. S5a† shows the schematic of the *in situ* fabrication of a photodetector device based on nanoparticles under light irradiation of different wavelengths, and the typical low magnification TEM image of the CdS nanoparticle photoelectric device is represented in Fig. S5b.† The structure composed of Cu, W metal electrodes, and CdS nanoparticles forms a complete device. Fig. S5c† shows the current–voltage curves of the photodetector device under light-on and -off conditions. The current shows an obvious gain under light exposure at the same voltage; this indicates that the photoelectric device can be successfully operated in the TEM using our homemade photoelectric holder.

## Conclusions

In summary, a special TEM specimen holder was designed, manufactured, and tested. Based on a commercially available STM-TEM holder, the custom-designed sample holder enabled *in situ* photoelectric characterization. The unique control of the power input for the light source prevented the light source from influencing the microscope performance while illuminating the sample with light of different wavelengths covering this entire visible spectrum. Using the movable probe, photoelectric nano-devices were successfully fabricated *in situ* during TEM imaging, and photocurrents on the pico-ammeter scale were detected while simultaneously imaging the crystal structure at the atomic level. This technique opens up the possibility to develop simultaneous *in situ* electrical, optical, and structural measurements and manipulation of the samples inside TEM without sacrificing the image quality. Our novel probe should be particularly useful to obtain a direct correlation between the structure at the atomic level and the performance of optically active materials and devices, namely, photovoltaic cells, quantum dots, photo resistors, and nanomaterials. Moreover, the new information thus obtained can trigger the development of new materials and devices.

## Conflicts of interest

There are no conflicts to declare.

## Supplementary Material

RA-008-C7RA10696C-s001

RA-008-C7RA10696C-s002

RA-008-C7RA10696C-s003

RA-008-C7RA10696C-s004

RA-008-C7RA10696C-s005

RA-008-C7RA10696C-s006

RA-008-C7RA10696C-s007
