# Supplementary material for: Simultaneous atomic-level visualization and high precision photocurrent measurements on photoelectric devices by in situ TEM
Source: RSC Adv. 2018 Jan 3;8(2):948–53. doi: 10.1039/c7ra10696c (PMC9077018; doi:10.1039/c7ra10696c)
Supplement: RA-008-C7RA10696C-s007 [file RA-008-C7RA10696C-s007.pdf]

Electronic Supplementary Material (ESI) for RSC Advances.
This journal is © The Royal Society of Chemistry 2017

Supporting Information for

Simultaneous Atomic-Level Visualization and High Precision Photocurrent Measurements on Photoelectric Devices by In situ TEM

Hui Dong[a], Tao Xu[a], Ziqi Sun[b], Qiubo Zhang[a], Xing Wu[d], Longbing He[a], Feng Xu[a], Litao Sun[a,c]**

a. SEU-FEI Nano-Pico Center, Key Laboratory of MEMS of Ministry of Education, Collaborative Innovation Center for Micro/Nano Fabrication, Device and System, Southeast University, Nanjing 210096, China

b. School of Chemistry, Physics and Mechanical Engineering, Queensland University of Technology, Gardens Point, Brisbane, QLD 4000, Australia

c.Southeast University-Monash University Joint Research Institute, Suzhou 215123, P. R. China

d. Department of Electrical Engineering, East China Normal University, 500 Dongchuan Road, Shanghai 200241, China

* fxu@seu.edu.cn; slt@seu.edu.cn.

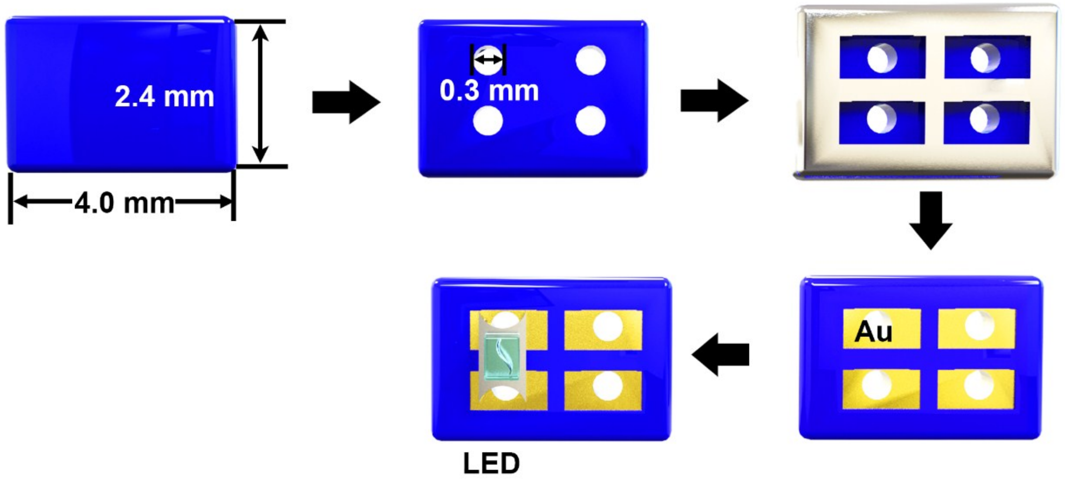

Fig. S1 The preparation process of LED as the light source on the holder. Sapphire was selected as the substrate serving as support for LED and electrode because of its high resistivity (>1011Ω·cm) in order to eliminate the influence of the LED current. There were the following steps on lightening the LED: Firstly, the sapphire was cut into the blocks in the size of 4.0 mm*2.4 mm rectangle substrate owing to restriction size of slot in the STM-TEM holder. Secondly, the holes with diameter of 0.3 mm were perforated on the substrate prepared for electrode support. Thirdly, the stainless shadow mask with corresponding size was placed on the substrate. Fourthly, Au thin film was deposited on the substrate by magnetron sputtering method. Finally, LED was welded on sputtering region with silver colloid at room temperature.

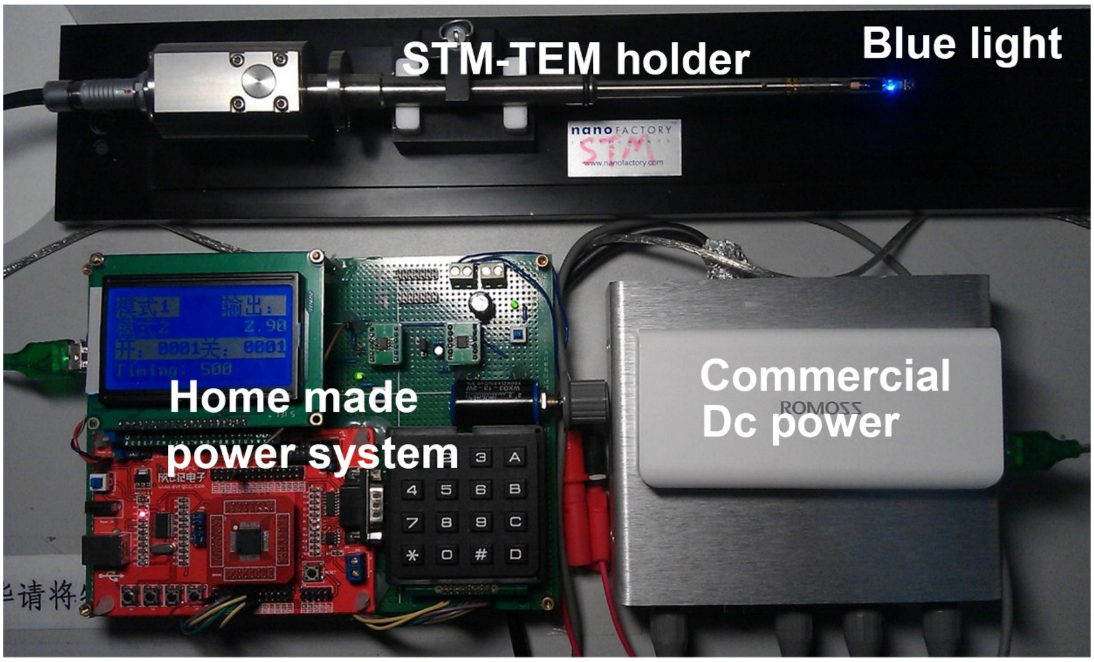

Fig. S2 The picture of the actual photoelectric holder with blue light on.

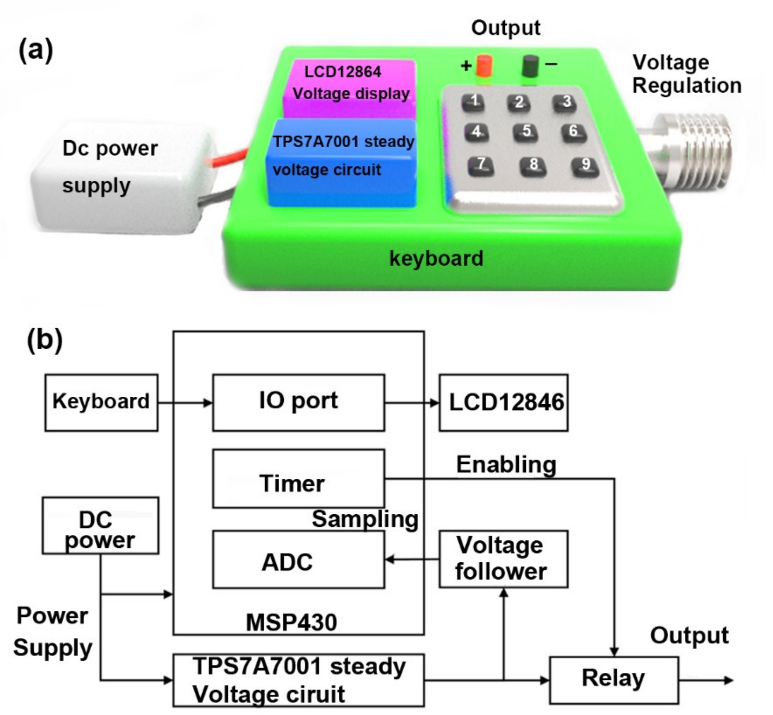

Fig. S3 (a) The schematic diagram of our elaborately designed power supply system for the LED. The precisely controller of voltage can be minimized as 0.01 V. (b) The working process of the system.

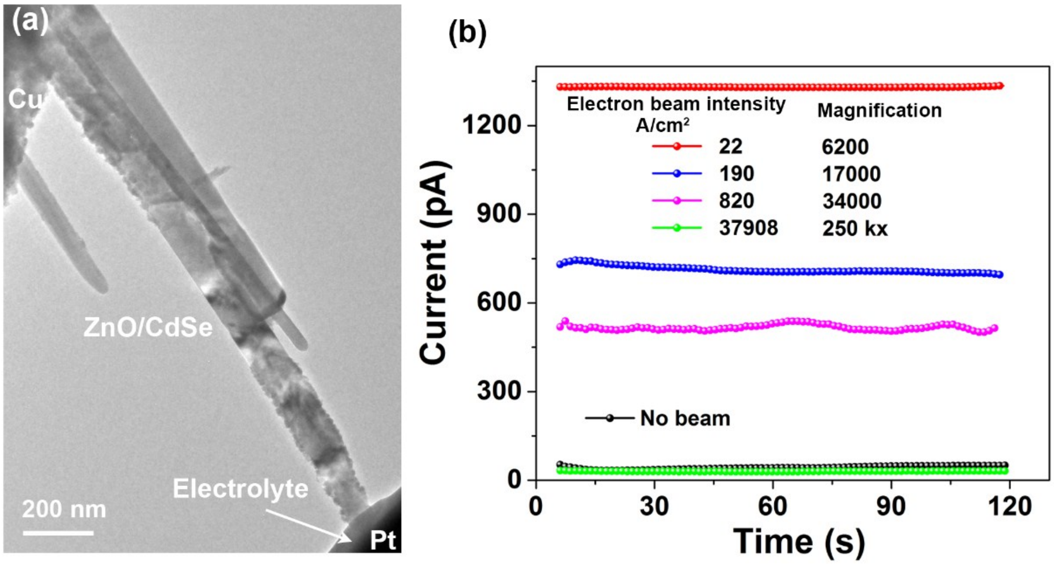

Fig.S4 (a) TEM image of one QDSSCs sample base on ZnO/CdSe. (b) The corresponding of electron beam induced current of different electron beam intensity.

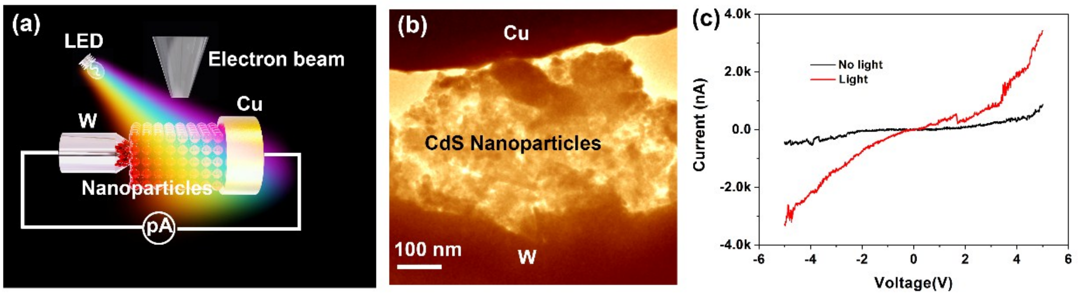

Fig. S5 (a) The schematic diagram of in-situ fabrication of photodetector device based on CdS nanoparticles under different wavelength. (b) TEM image of CdS nanoparticles photodetector device. (c) Current-voltage (I-V) characteristics with white light on and off. The current shows a large gain under light exposure.

Code S1: Program code of C language to control the power supply system and regulate output voltage.

```
#include <msp430x26x.h>
#include<stdlib.h>
#include "Define.h"
#include "Cry12864.h"
#include "Cry12864.c"
#include "key_4x4.c"
/*******Save ADC conversion result array*****/
#define     Num_of_Results     64//The number of times the ADC samples used to calculate the average value
static uint results[Num_of_Results];
unsigned long sum;//32 times the sum of the sampled values


/************system status************/
uchar SetState=1;//Set the state, 1 means that the various parameters can be modified


/************Input the key value************/
extern uchar keyvalue;



/******** Fixed a display string **********/
uchar row1[]="SMDLEDpower";
uchar row2[]="defaut：";
uchar row3[]="measured：";
uchar row4[]="mA";
uchar row5[]="Step value：";
uchar ErrorMessage[]="Error!";
/******** Cursor position array ************/
uchar CursorAddr[3]={0x95,0x96,0x9e};
uchar Cur=0;
/********* The number of values entered at the cursor level ************/
uchar InputState=0;
/********* Preset current value, measured current value and step value ************/
int CurrentGet=0;
int CurrentSet=0;
int CurrentStep=0;
uchar CurrentGet_Char[5]={0,0,0,0,'\0'};
uchar CurrentSet_Char[5]={0,0,0,0,'\0'};//Current preset value
uchar CurrentStep_Char[3]={0,0,'\0'};
/*********10mA~20mA DAC Register value************/
```

```
uchar CurrentPre[]={5,7,9,10,12,14,15,17,19,21};
uchar nBitmapDot[] =                              // data sheet
{
        0x00,0x00,0x18,0x00,0x00,0x00,0x3C,0x00,
        0x00,0x00,0xFC,0x00,0x00,0x01,0xF0,0x00,
        0x00,0x03,0xC0,0x00,0x00,0x03,0x80,0x00,
        0x00,0x03,0x80,0x00,0x00,0x03,0x80,0x00,
        0x00,0x03,0x80,0x00,0x00,0x03,0x80,0x00,
        0x00,0x03,0x80,0x00,0x00,0x03,0x80,0x00,
        0x00,0x03,0x80,0x00,0x00,0x03,0x80,0x00,
        0x00,0x03,0x80,0x00,0x00,0x03,0x80,0x00,
        0x00,0x0F,0xF0,0x00,0x00,0x7F,0xFE,0x00,
        0x00,0xFF,0xFF,0x00,0x03,0xF0,0x0F,0xC0,
        0x07,0xC0,0x03,0xE0,0x0F,0x00,0x00,0xF0,
        0x1E,0x00,0x00,0x78,0x1C,0x00,0x00,0x3C,
        0x38,0x00,0x00,0x1C,0x78,0x00,0x00,0x1E,
        0x70,0x00,0x00,0x0E,0x70,0x00,0x00,0x0E,
        0xE0,0x00,0x00,0x07,0xE0,0x00,0x00,0x07,
        0xFF,0xFF,0xFF,0xFF,0xFF,0xFF,0xFF,0xFF,
        0xFF,0xFF,0xFF,0xFF,0xE0,0x00,0x00,0x07,
        0xE0,0x00,0x00,0x07,0xE0,0x00,0x00,0x07,
        0x70,0x00,0x00,0x0E,0x70,0x00,0x00,0x0E,
        0x78,0x00,0x00,0x1E,0x38,0x00,0x00,0x1C,
        0x1C,0x00,0x00,0x3C,0x1E,0x00,0x00,0x78,
        0x0F,0x00,0x00,0xF0,0x07,0xC0,0x03,0xE0,
        0x03,0xF0,0x0F,0xC0,0x01,0xFF,0xFF,0x80,
        0x00,0x7F,0xFE,0x00,0x00,0x0F,0xF0,0x00,
        0x00,0x01,0xC0,0x00,0x00,0x01,0xC0,0x00,
        0x00,0x01,0xC0,0x00,0x00,0x01,0xC0,0x00,
        0x00,0x01,0xC0,0x00,0x00,0x01,0xC0,0x00,
        0x00,0x01,0xC0,0x00,0x00,0x01,0xC0,0x00,
        0x00,0x01,0xC0,0x00,0x00,0x01,0xC0,0x00,
        0x00,0x01,0xC0,0x00,0x00,0x03,0xC0,0x00,
        0x00,0x07,0xC0,0x00,0x00,0x0F,0x00,0x00,
        0x00,0x3E,0x00,0x00,0x00,0x3C,0x00,0x00
};
/******************************************
Name of function: Save Temp Data
Function: Temporarily save the input preset current value or step value******************************************/
void SaveTempData()
{
    if(Cur==0x02)
        CurrentStep_Char[InputState]=keyvalue;// Save the step value to the string
```

```
else
      {
        CurrentSet_Char[Cur*2+InputState]=keyvalue;//
      }
}
/*******************************************
Name of function:GetCursorAddr
Features: Get the cursor position
*******************************************/
uchar GetCursorAddr(uchar Curs)
{
   if(Curs==0x03) Cur=0x00;
   if(Curs==0xff) Cur=0x02;
   return CursorAddr[Cur];
}

/*******************************************
Name of function: NumToChar
Features: Converts a number to a 4-digit string
*******************************************/
void NumToChar(int x,uchar *y)
{
   *y=x/1000+0x30;
   *(++y)=(x/100)%10+0x30;
   *(++y)=(x/10)%10+0x30;
   *(++y)=x%10+0x30;
}
/*******************************************
Name of function:InitDAC
Function: DAC Module initialization
*******************************************/
void InitDACandADC()
{
   ADC12CTL0 = REF2_5V + REFON;                    // Internal 2.5V ref on

   TACCR0 = 13600;                                 // DELAY to allow Ref to settle
   TACCTL0 |= CCIE;                                // Compare-mode interrupt.
   TACTL = TACLR + MC_1 + TASSEL_2;                // up mode, SMCLK
   __bis_SR_register(LPM0_bits + GIE);             // Enter LPM0, enable interrupts
   TACCTL0 &= ~CCIE;                               // Disable timer interrupt
   //__disable_interrupt();                        // Disable Interrupts
   DAC12_1CTL = DAC12CALON + DAC12IR + DAC12AMP_5 + DAC12ENC;//DAC12SREF_2; // Int ref gain 1
   DAC12_1DAT = 0x0000;                            // 1.0V (2.5V = 0x0FFFh)，DAC12_1
```

output on P6.7

```
   P6SEL |= BIT0;                                       // Enable the ADC channel,P6.0
   ADC12CTL0 |= ADC12ON+SHT0_12+MSC;                  // Open ADC， Set the sampling time
   ADC12CTL1 = CSTARTADD_0+SHP+CONSEQ_2+ADC12SSEL_1;        // Select the memory
address and use the sampling timer
   ADC12MCTL0 = INCH_0+SREF_1;                                 // The memory 7 selects the
sampling channel 3
   ADC12IE = BIT0;                                      // Enable ADC interrupt
   ADC12CTL0 |= ENC;                                    // ADC12 enable
   ADC12CTL0 |= ADC12SC;                                 // ADC12 Start sampling and convert
}
#pragma vector = TIMERA0_VECTOR
__interrupt void TA0_ISR(void)
{
  TACTL = 0;                                            // Clear Timer_A control registers
   __bic_SR_register_on_exit(LPM0_bits);         // Exit LPMx, interrupts enabled

}
/******************************************
Name of function：ADC12ISR
Function：ADC Interrupt service function， Here   multiple times of averaged value is used to
            calculate    the    analog    voltage    value    of    the    port    P6.7
******************************************/
#pragma vector=ADC12_VECTOR
__interrupt void ADC12ISR (void)
{
   static uint index = 0;
   results[index++] = ADC12MEM0;                        // Move results
   if(index == Num_of_Results)
   {
         uchar i;
         sum = 0;
         index = 0;
         for(i = 0; i < Num_of_Results; i++)
         {
               sum += results[i];
         }
         sum >>= 6;                                     // Divided by 32
         if(CurrentSet>=10&&CurrentSet<20)//Output current value 10mA~20mA
               CurrentGet=(int)(sum*259/464+7.7263);
          else if(CurrentSet>=100)
         CurrentGet=(int)(sum/1.67374+6.6022);// The output current is greater than 100mA
```

```
            else
               CurrentGet=(int)(sum/1.675+6.67164);
         NumToChar(CurrentGet,CurrentGet_Char);
         DispMultiChar(0x8d,CurrentGet_Char,4);
   }
}

void main()
{
    WDTCTL = WDTPW + WDTHOLD;

    BCSCTL1 &= ~XT2OFF;                        // Turn on the XT2 high frequency crystal
oscillator
    BCSCTL2 |=SELM_2+SELS;
    BCSCTL3 |= XT2S_2;                          // 3 ?16MHz crystal or resonator
    while((OFIFG&IFG1)==BIT1)
    {
       IFG1&=~BIT1;
       for(uchar p=0;p<0xff;p++);
    }

    P1DIR=0xff;
    Ini_Lcd();
    InitKeyvalue();
    Clear_GDRAM();
    Disp_HZ(0x82,row1,5);
    Disp_HZ(0x92,row2,3);
    Disp_HZ(0x8a,row3,3);
    Disp_HZ(0x9a,row5,4);
    DispMultiChar(0x9f,row4,2);
    DispMultiChar(0x97,row4,2);
    DispMultiChar(0x8f,row4,2);
    Draw_32x64(nBitmapDot);
    Write_Cmd(DISPLAY_ON_CursorON);
    NumToChar(CurrentSet,CurrentSet_Char);
    DispMultiChar(0x95,CurrentSet_Char,4);
    Write_Cmd(CursorAddr[Cur]);
    InitDACandADC();
    __disable_interrupt();
    while(1)
    {
       GetKeyvalue();
       switch(keyvalue)
    {
```

```
        case'A':// The cursor moves up and the number of inputs is set to zero              if(!SetState)
break;// The settings are closed and the parameters can not be modified
Write_Cmd(GetCursorAddr(--Cur));
          InputState=0;
          break;
        case'B':// The cursor moves down and the number of inputs is set to zero              if(!SetState)
break;// The settings are closed and the parameters can not be modified
          Write_Cmd(GetCursorAddr(++Cur));
          InputState=0;
          break;
        case'1':case'2':case'3':case'4':case'5':case'6':case'7':case'8':case'9':case'0':
          if(!SetState) break;// The settings are closed and the parameters can not be modified
          SaveTempData();//Save the input data to the temporary string according to the current
cursor and the number of entries
          Write_Data(keyvalue);
          InputState++;
          if(InputState==2)// Enter the two digits, the cursor jump to the next weapon input number
to 0
          {
            Write_Cmd(GetCursorAddr(++Cur));
            InputState=0;
          }
          break;
         case'*':// Decrease by step value
            if(SetState) break;// Setting is on and stepping can not be used
            CurrentSet-=CurrentStep;
             if(CurrentSet>2100||CurrentSet<10)
           {
             DispMultiChar(0x95,ErrorMessage,6);
             delay_ms(1500);
             CurrentSet=10;
             DAC12_1DAT=(int)(CurrentPre[0]);
             DispMultiChar(0x97,row4,2);
           }
           else if(CurrentSet>=10&&CurrentSet<20)
               DAC12_1DAT=(int)(CurrentPre[CurrentSet-10]);
           else if(CurrentSet>=100)
             DAC12_1DAT=(int)(CurrentSet*1.675-12.5);
           else
             DAC12_1DAT=(int)(CurrentSet*1.6875-11.75);
           // Display the set current value
             NumToChar(CurrentSet,CurrentSet_Char);
             DispMultiChar(0x95,CurrentSet_Char,4);
             __enable_interrupt();
```

```
        break;
      case'#':// Increase by step value
        if(SetState) break;// Setting is on and stepping can not be used
CurrentSet+=CurrentStep;
         if(CurrentSet>2100||CurrentSet<10)
        {
          DispMultiChar(0x95,ErrorMessage,6);
          delay_ms(1500);
          CurrentSet=10;
          DAC12_1DAT=(int)(CurrentPre[0]);
          DispMultiChar(0x97,row4,2);
        }
        else if(CurrentSet>=10&&CurrentSet<20)
             DAC12_1DAT=(int)(CurrentPre[CurrentSet-10]);
        else if(CurrentSet>=100)
          DAC12_1DAT=(int)(CurrentSet*1.675-12.5);
        else
          DAC12_1DAT=(int)(CurrentSet*1.6875-11.75);
        // Displays the set current value
          NumToChar(CurrentSet,CurrentSet_Char);
          DispMultiChar(0x95,CurrentSet_Char,4);
          __enable_interrupt();
        break;
      case'C':// OK key is set to save the final result
        SetState=0;// Close the settings
        Write_Cmd(GetCursorAddr(0x00));// Set the cursor
        InputState=0;// Enter the number of settings 0
        CurrentSet=atoi((char*)CurrentSet_Char);
        CurrentStep=atoi((char*)CurrentStep_Char);
        Write_Cmd(DISPLAY_ON_CursorOFF);//Close the cursor
        if((CurrentSet>2100)||(CurrentSet<10))
        {
          DispMultiChar(0x95,ErrorMessage,6);
          delay_ms(1500);
          CurrentSet=10;
          DAC12_1DAT=(int)(CurrentPre[0]);
          DispMultiChar(0x95,"0010",4);
          DispMultiChar(0x97,row4,2);
        }
        else if(CurrentSet>=10&&CurrentSet<20)
             DAC12_1DAT=(int)(CurrentPre[CurrentSet-10]);
        else if(CurrentSet>=100)
          DAC12_1DAT=(int)(CurrentSet*1.675-12.5);
        else
```

```
        DAC12_1DAT=(int)(CurrentSet*1.6875-11.75);
      __enable_interrupt();
      break;
    case'D':// Set the key, you can modify the various parameters
      SetState=1;//Open the settings
      Write_Cmd(DISPLAY_ON_CursorON);//Turn the settings
      Write_Cmd(GetCursorAddr(0x00));//Setting the cursor
      InputState=0;// The number of inputs is set to zero
      break;
  }
  }
}
```
